# Characterization of MK6240, a tau PET tracer, in autopsy brain tissue from Alzheimer’s disease cases

**DOI:** 10.1007/s00259-020-05035-y

**Published:** 2020-09-24

**Authors:** Mona-Lisa Malarte, Agneta Nordberg, Laetitia Lemoine

**Affiliations:** 1 Department of Neurobiology Care Sciences and Society, Division of Clinical Geriatrics, Center of Alzheimer Research, Karolinska Institutet, Stockholm, Sweden; 2 Theme Aging, Karolinska University Hospital, Stockholm, Sweden

**Keywords:** Alzheimer’s disease, Tau imaging, Autoradiography, MK6240, AV-1451, THK5117

## Abstract

**Purpose:**

MK6240 is a second-generation tau PET tracer designed to detect the neurofibrillary tangles in the brains of patients with Alzheimer’s disease (AD). The aim of the study was to characterize ^3^H-MK6240 in AD and control brain tissue and to compare its binding properties with those of first-generation tau PET tracers.

**Methods:**

Saturation binding assays with ^3^H-MK6240 were carried out in the temporal and parietal cortices of AD brains to determine the maximum number of binding sites (Bmax) and the dissociation constants (Kd) at these sites. Competitive binding assays were carried out between ^3^H-MK6240 and unlabelled MK6240, AV-1451 (aka T807, flortaucipir) and THK5117, and between ^3^H-THK5351 and unlabelled MK6240. Regional binding studies with ^3^H-MK6240 were carried out in homogenates from six AD and seven control brains and, using autoradiography, on large frozen sections from two AD brains and one control brain.

**Results:**

The saturation binding assays gave Bmax and Kd values of 59.2 fmol/mg and 0.32 nM in the temporal cortex and 154.7 fmol/mg and 0.15 nM in the parietal cortex. The competitive binding assays revealed two binding sites with affinities in the picomolar and nanomolar range shared by ^3^H-MK6240 and all the tested unlabelled compounds. There were no binding sites in common between ^3^H-THK5351 and unlabelled MK6240. Regional binding of ^3^H-MK6240 was significantly higher in AD brain tissue than in controls. Binding in brain tissue from AD patients with early-onset AD was significantly higher than in brain tissue from patients with late-onset AD. Binding of ^3^H-MK6240 was not observed in off-target regions**.** Autoradiography showed high regional cortical binding in the two AD brains and very low binding in the control brain.

**Conclusions:**

^3^H-MK6240 has a high binding affinity for tau deposits in AD brain tissue but also has different binding characteristics from those of the first-generation tau tracers. This confirms the complexity of tau tracer binding on tau deposits with different binding affinities for different binding sites.

**Electronic supplementary material:**

The online version of this article (10.1007/s00259-020-05035-y) contains supplementary material, which is available to authorized users.

## Introduction

Alzheimer’s disease (AD) is characterized by the extracellular amyloid beta accumulation in the form of amyloid plaques and abnormal intracellular accumulation of tau protein into neurofibrillary tangles (NFTs) in the brain, both of which lead to progressive neurodegeneration [1]. The abnormal accumulation of tau protein is a characteristic of several other diseases which can be grouped under the generic name tauopathies [2]. The range of clinical phenotypes of these tauopathies can make clinical diagnosis very challenging [3], and there is a need for new tools to help understand and differentiate between them in vivo in the early stages of the disease.

PET tracers for studying the progression of tau deposits in vivo have been synthesized. However, this has been challenging because the deposits are also intracellular: the tau PET tracers need to be able to cross both the blood-brain barrier and the cell membrane. Moreover, the method of tau inclusion varies with the tauopathy; exon 10 may be included or excluded during the alternative splicing of the tau gene, leading to three-repeat (3R) or four-repeat (4R) tau isoforms [4]. The first generation of tau PET tracers, including THK family compounds and AV-1451 (also known as T807, flortaucipir), has been widely explored in vivo in AD patients, and its effects have been compared with those in controls; this has generated important information about the progression of tau pathology and its relation to functional changes in patients (see reviews [5–7]). In vitro characterization of the first-generation tracers has also been extensively carried out. In vitro binding assays have indicated that ^3^H-THK5117 has good binding properties, with high affinity and specificity for tau in post-mortem brain tissue from AD patients [8]. Using autoradiography techniques and AT8 immunostaining on adjacent paraffin sections from an AD brain, we have previously observed similar regional distributions of ^3^H-THK5117 and tau [8]. Binding assays and competitive studies of first-generation tau PET tracers indicated that THK5117, THK5351 and AV-1451 targeted the same binding sites with different affinities, whereas PBB3 seemed to have its own target [9, 10]. In 2017, Fitzpatrick et al. used cryo-electron microscopy to describe the structure of tau fibrils. In silico studies using this structure have suggested the presence of multiple theoretical binding sites for which the first generation of tau tracers had differing affinities [11]. Unfortunately, first-generation tau PET tracers also demonstrated off-target binding: THK5117, THK5351 and AV-1451 bind to monoamine oxidase B (MAO-B) [12], AV-1451 binds to neuromelanin [13] and PBB3 binds to amyloid plaques and alpha-synuclein [14, 15]. To avoid this scenario, a second generation of tau PET tracers has been designed; these include RO948, PI2620, JNJ311, MK6240, PM-PBB3 and AM-PBB3 [16]. The results of preliminary studies are promising for many of these. RO948 has good in vivo kinetic distribution and brain uptake, with low off-target binding [17]; PI2620 has a high affinity for pathological tau aggregates and has entered clinical trials [18]; results for JNJ311 are promising in trials of rodent and non-human primate tissue [19]; MK6240 binds specifically to one site on NFT-rich AD brain tissue and neither binds to off-target sites nor has an affinity for amyloid plaques [20–22]. In silico data have suggested that MK6240, JNJ311 and PI2620 have a low affinity for MAO-B [23].

An in vitro study of the second-generation MK6240 tracer has shown a high binding affinity for brain homogenate rich in NFTs [21, 24]. Similarly, a recent in vivo pilot study by Lohith et al. in AD patients showed that MK6240 binding occurred in regions rich in NFT deposits [25]; More recently, Pascoal et al. reported that ^18^F-MK6240 could be used to differentiate early and late disease stages of AD as well as discriminating AD from cognitively unimpaired and frontotemporal dementia [26]. In silico modelling has suggested the possibility of four theoretical binding sites on tau fibrils for MK6240 [27].

In this study, our aim was to characterize the ^3^H-MK6240 binding pattern in human autopsy brain tissue from AD cases and to compare this with binding in brain tissue from age-matched controls. We used binding assays in brain homogenates and autoradiography on large frozen hemispherical sections and compared the results with the binding properties of several first-generation tau PET tracers.

## Materials and methods

### Chemicals

^3^H-MK6240 (specific activity (SA) = 45.77 Ci/mmol) and unlabelled MK6240 were provided by Merck Sharp and Dohme Corp. (Whitehouse Station, USA). ^3^H-THK5351 (SA = 35 Ci/mmol) was synthesized and labelled at the Centre for Psychiatric Research, Department of Clinical Neuroscience, Karolinska Institutet (Solna, Sweden). Unlabelled AV-1451 was synthesized by He Tian (Institute of Fine Chemicals, East China University of Science and Technology, Shanghai, China). Unlabelled THK5117 was custom synthesized by Novandi (Södertalje, Sweden).

### Brain tissue

Frozen post-mortem tissue from eight AD and seven control brains from the Netherlands Brain Bank (NBB) was homogenized in phosphate buffer saline (PBS) with additional 0.1% bovine serum albumin (BSA; 250 mg/mL) and protease/phosphatase inhibitors (10 μL/mL).

Large hemispherical frozen post-mortem brain sections (100 μm thick) were obtained from two patients with AD who had been clinically followed by one of the authors (A.N.) at the Department of Geriatric Medicine, Karolinska University Hospital Huddinge, Sweden, and one control.

Table 1 provides a summary of demographic information about the patients and controls from which brain tissue was sourced.

**Table 1:** Demographic information about the patients and controls from whom brain tissue was obtained for binding assays (AD case numbers 1–8) and autoradiography studies (AD patients A and B and control C). The sex, age at death, apolipoprotein (ApoE) genotype, Braak stage at cerebral pathological assessment, presence of early- or late-onset AD and post-mortem delay are provided

	Case no.	Sex (M/F)	Age (years)	ApoE (E/E)	Braak stage	EOAD/LOAD	Post-mortem delay (hours:minutes)
AD patients	*1*	*F*	*59*	*4/4*	*5*	*EOAD*	*04:20*
	2	F	66	4/3	5	EOAD	06:30
	3	M	70	4/4	4	EOAD	04:00
	*4	*M*	*77*	*NA*	*6*	*LOAD*	*06:35*
	*5*	*M*	*78*	*4/4*	*5*	*LOAD*	*06:35*
	6	F	81	4/3	5	LOAD	06:15
	*7	F	82	NA	6	LOAD	04:20
	*8	F	85	3/3	4	LOAD	06:00
	Mean	3 M/5F	72 ± 13	5 E4 1 E3	4–6	3 EOAD/5 LOAD	
	A	F	79	4/4	5	LOAD	16:00
	B	M	81	4/4	6	LOAD	17:00
Controls	9	F	50	3/3	1	N/A	04:10
	10	M	62	3/3	1	N/A	07:20
	11	F	71	3/3	1	N/A	07:10
	12	F	77	3/3	1	N/A	02:55
	13	M	78	3/3	1	N/A	17:40
	14	M	79	3/3	2	N/A	09:00
	15	F	84	3/3	1	N/A	06:55
	Mean	3 M/4F	67 ± 17	7 E3 0 E4	1–2		
	C	F	76		1	*N/A*	*04:00*

Values in italics indicate AD patients (numbers 1, 4, 5) whose temporal lobes were used for the saturation and competition studies, and the 59-year-old AD patient (number 1) whose parietal lobe was used. The asterisks ‘*’ indicate AD patients (numbers 4, 7, 8) whose hippocampus was used for the competition studies

*AD*, Alzheimer’s disease; *EOAD*, early-onset AD; *F*, female; *LOAD*, late-onset AD; *M*, male; *N/A*, not applicable

### Binding assays

Optimal binding assay conditions were established for ^3^H-MK6240 by varying the quantity of the brain homogenate (0.2, 0.5, 0.75, 1 or 2 mg/mL) and the incubation temperature (25 °C, 37 °C) (see Supplemental Fig. 1).

Saturation binding assays were carried out using homogenates of the temporal and parietal cortices from AD cases (0.2 mg/mL), with increasing concentrations of ^3^H-MK6240 (0.05–2 nM) incubated for 90 min at 37 °C in PBS + 0.1% BSA (pH 7.4). The extent of non-specific binding was determined using 1 μM unlabelled MK6240. The binding reaction was terminated by filtering the sample through glass fibre filters (which had been pre-soaked for at least 3 h in 0.3% polyethylenimine solution) and washing it three times with cold binding assay buffer. The extent of ^3^H-MK6240 binding was quantified using a scintillation counter (Beckman Coulter LS6500). The equilibrium dissociation constant (Kd) and the maximum number of binding sites (Bmax) were determined using GraphPad Prism software version 8 for Mac OSX.

Competitive binding assays with ^3^H-MK6240 (0.5 nM) were carried out for 90 min at 37 °C on temporal cortex homogenates from two AD brains, in PBS + 0.1% BSA (pH 7.4), using increasing concentrations of unlabelled MK6240, unlabelled THK5117 or unlabelled AV-1451 (1.10^−14^–1.10^−5^ M). The binding assay was terminated as described above. The half-maximal effective concentration (IC50) was determined using GraphPad Prism software and data were analysed using a 2-site competition model.

Competitive binding assays were also carried out with ^3^H-THK5351 (1.5 nM) in PBS + 0.1% BSA (pH 7.4) on hippocampus homogenates, using increasing concentrations of unlabelled MK6240 (1.10^−14^–1.10^−5^ M) in PBS + 0.1% BSA for 2 h at room temperature.

The regional distribution of ^3^H-MK6240 (0.5 nM) to seven brain regions (frontal, temporal and parietal cortices, hippocampus, thalamus, caudate nucleus and cerebellum) was investigated using tissue from six AD patients (three with early-onset AD (EOAD, <65 years old) and three with late-onset AD (LOAD, > 65 years old)) and seven age-matched controls. The extent of non-specific binding was determined using unlabelled MK6240 (1 μM). Data were analysed using GraphPad Prism software. ^3^H-MK6240 binding in AD brain tissue was compared with that in control brain tissue using a 2-way ANOVA test with Graphpad Prism 8 software.

### Autoradiography binding studies in Alzheimer’s disease and control autopsy brain tissue

A regional distribution study of ^3^H-MK6240 using autoradiography on large frozen brain sections was also carried out. After being allowed to dry, the sections were pre-incubated for 15 min in PBS + 0.1% BSA (pH 7.4) before incubation for 1 h with ^3^H-MK6240 (0.5–1 nM) at room temperature. The extent of non-specific binding was determined using unlabelled MK6240 (1 μM). After 1 h, the sections were washed three times for 5 min in cold binding assay buffer (4 °C) and briefly dipped into cold de-ionized water (4 °C). After overnight drying, they were put into a phospho-image cassette with a phospho-plate apposed onto the sections for 7 days, with a tritiated standard. The plates were then scanned with the BAS-2500 phosphor imager and regions of interest were drawn manually using multigauge software.

## Results

**Fig. 1:**
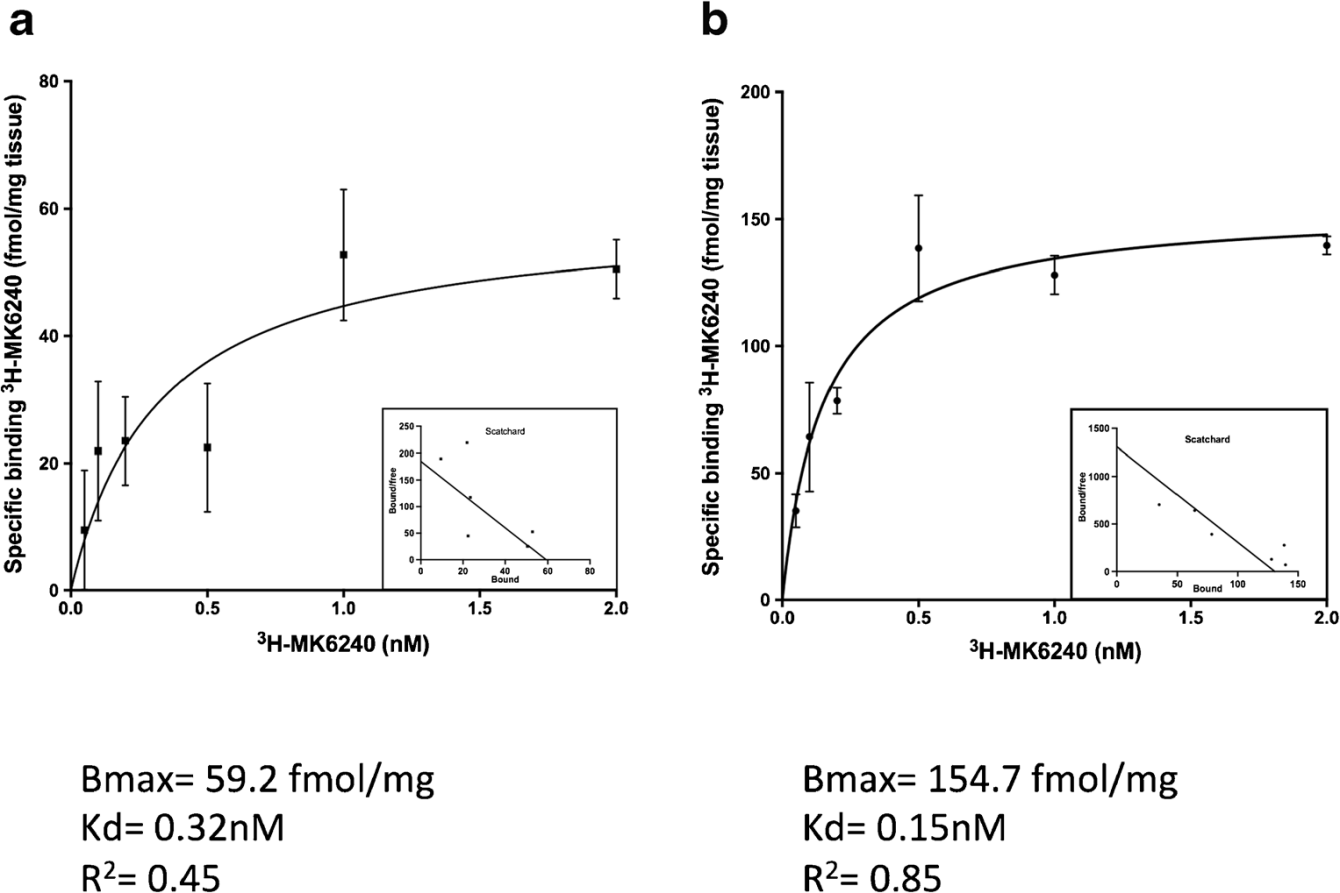
Saturation of binding sites in the temporal and parietal cortices of patients with Alzheimer’s disease (AD). **a** Saturation curve for ^3^H-MK6240 (0.05–2 nM) in temporal cortex brain homogenates from two AD patients (patients 4 and 5). The solid regression line was determined by Graphpad Prism software. Analyses using GraphPad Prism software showed one binding site. **b** Saturation curve for ^3^H-MK6240 (0.05–2 nM) in the parietal cortex brain homogenate from one AD patient (patient 1). Bmax = maximum number of binding sites; Kd = dissociation constant; *R*^2^ = regression coefficient

Saturation binding assay results for the AD brain tissue are shown in Fig. 1. The mean saturation binding curve in the temporal cortices of two AD cases is shown in Fig. 1a. Using a ^3^H-MK6420 concentration range from 0.05 to 2 nM, we observed a Bmax of 59.2 fmol/mg and a Kd of 0.32 nM. In the same experiment carried out in the parietal cortex of one AD brain (case 1), we observed a Bmax of 154.7 fmol/mg and a Kd of 0.15 nM. Data transformed in a Scatchard plot suggested one binding site in both regions.

**Fig. 2:**
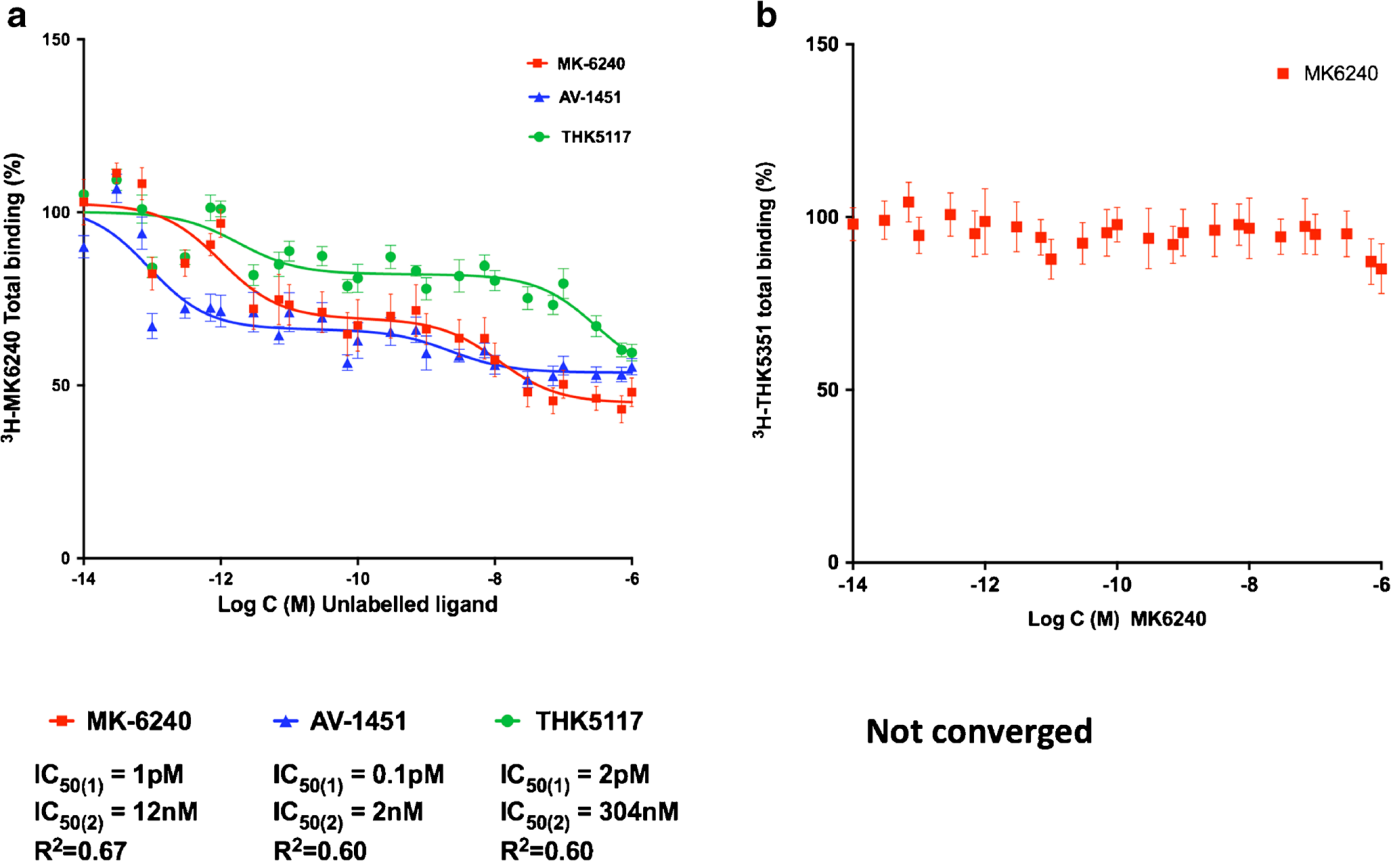
**a** Competitive binding assays between ^3^H-MK6240 (0.5 nM) and increasing concentrations of unlabelled MK6240, AV-1451 and THK5117 (1.10^−14^–1.10^−6^ M). **b** Competitive binding assay in hippocampus tissue from the brains of three patients with Alzheimer’s disease using ^3^H-THK5351 (1.5 nM) and increasing concentrations of MK6240 (1.10^−14^–1.10^−6^ M). Error bars represent the SEM from three experiments, each in triplicate, for each unlabelled compound. C = concentration; high = high-affinity binding site; IC50 = half-maximal effective concentration; low = low-affinity binding site; *R*^2^ = regression coefficient; supHigh = super-high-affinity binding site

The competitive binding assay between ^3^H-MK6240 and unlabelled MK6240 (Fig. 2a) showed two relevant binding sites: the unlabelled competitor had a super-high affinity for site 1, in the picomolar range: IC_50(supHigh)_ = 1 pM and a high affinity for site 2, in the nanomolar range: IC_50(High)_ = 12 nM. AV-1451 and THK5117 were also in competition with ^3^H-MK6240 at two binding sites: IC_50(supHigh)_ = 0.1 pM, IC_50(High)_ = 2 nM and IC_50(supHigh)_ = 2 pM, IC_50(low)_ = 304 nM, respectively. The proportion of binding sites for which the tracers had a super-high affinity (corresponding to IC50_(supHigh_) is shown in Table 2. In the competitive studies between unlabelled MK6240 and ^3^H-MK6240, the unlabelled competitor had a super-high affinity for 58% of the sites and a high affinity for 42% of the sites. AV-1451 had a super-high affinity for the highest proportion of sites (74%) when competing with ^3^H-MK6240, whereas the THK family compounds had the lowest values; THK5117 had a super-high affinity for 37% of the sites.

**Table 2:** Percentage of ^3^H-MK6240 binding sites in the temporal cortex (or ^3^H-THK5351 binding sites in the hippocampus) for which the unlabelled compound (THK5117, AV-1451 or MK6240) had a super-high affinity in competitive studies

Radioligand	Competitor	Fraction of sites with super-high affinity for the competitor (%)	*R*^2^
^3^H-MK6240	AV-1451	74	0.60
	THK5117	37	0.60
	MK6240	58	0.70
^3^H-THK5351	AV1451 *	35	0.96
	THK5117*	46	0.96
	MK6240	0	N/A

*See Lemoine et al. [12] for competitive studies using ^3^H-THK5351 versus unlabelled THK5117 or AV-1451

*N/A*, not applicable; *R*^*2*^, regression coefficient

Figure 2b shows the results of competitive binding studies using increasing concentrations of unlabelled MK6240 and ^3^H-THK5351. The unlabelled MK6240 did not compete with ^3^H-THK5351.

**Fig. 3:**
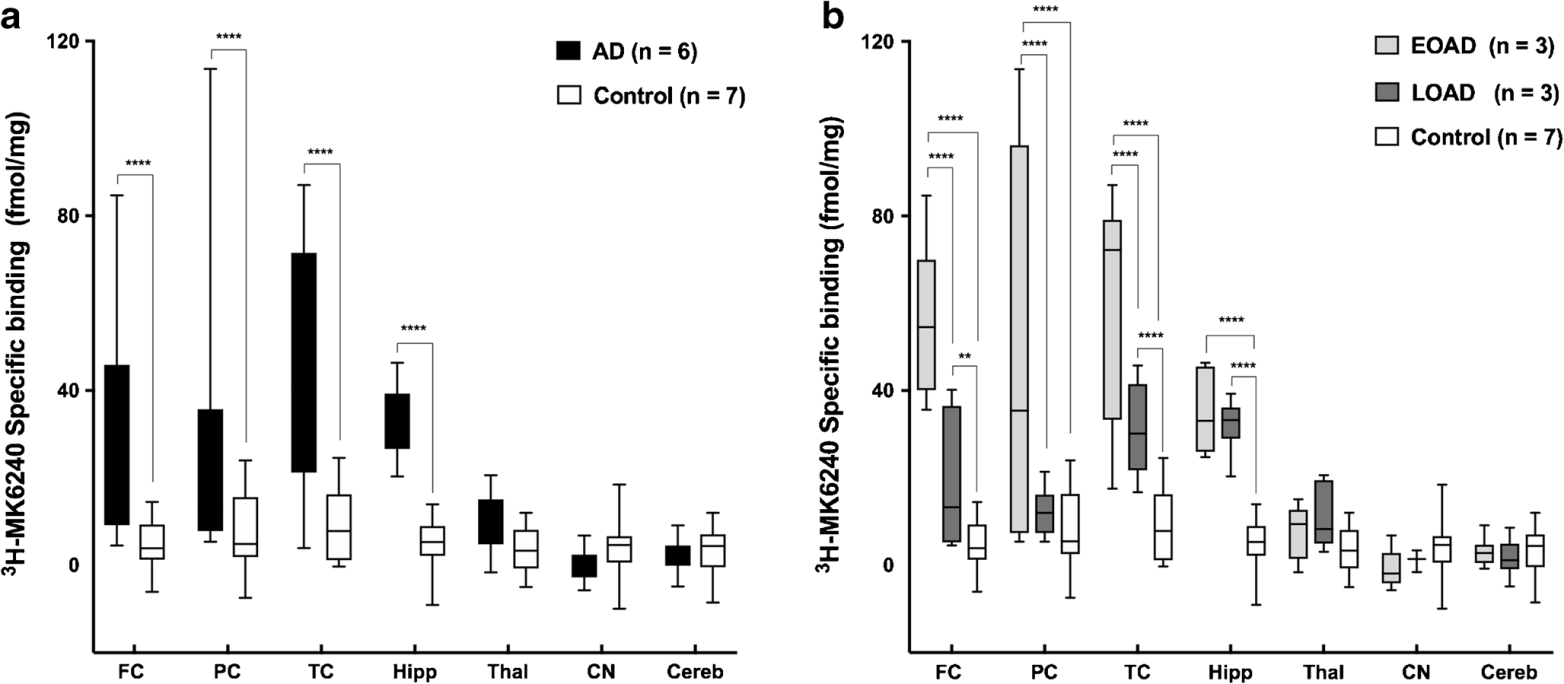
Regional binding distribution of ^3^H-MK6240 in post-mortem brain homogenates from **a** six cases with Alzheimer’s disease (AD) and seven controls and **b** the same six cases separated according to whether they had early-onset AD (EOAD; three cases) or late-onset AD (LOAD; three patients), with the same seven controls. The regions of interest were the frontal cortex (FC), parietal cortex (PC), temporal cortex (TC), hippocampus (Hipp), thalamus (Thal), caudate nucleus (CN) and cerebellum (Cereb). Error bars represent the SEM from three experiments, each in triplicate, for each area. Prism software was used for a 2-way analysis of variance multiple comparisons. ***p < 0.001, ****p < 0.0001

The regional distribution of ^3^H-MK6240 in AD and control brain homogenates is shown in Fig. 3. We detected low specific binding in the seven brain regions of all the control brains. ^3^H-MK6240 binding in the frontal, parietal and temporal cortices and hippocampus from AD brains was statistically significantly higher (*p* < 0.0001) than in these regions in control brains. No or low binding was observed in the thalamus, caudate nucleus and cerebellum in AD brains, with no significant differences from control brains (Fig. 3a). Figure 3b shows the regional distribution when the data were analysed according to whether the patients had EOAD or LOAD. The binding of ^3^H-MK6240 was significantly higher in EOAD brain tissue than in LOAD and control tissue in cortical regions (frontal, parietal and temporal cortices; *p* < 0.0001) with the highest binding occurring in the temporal cortex. The binding was significantly higher in the EOAD hippocampus than in the control hippocampus (*p* < 0.0001). The binding was also significantly higher in LOAD tissue than in control tissue for the frontal cortex (*p* = 0.0084), temporal cortex (*p* < 0.0001) and hippocampus (*p* < 0.0001). There were no differences between EOAD and LOAD binding in the subcortical regions (thalamus and caudate nucleus).

**Fig. 4:**
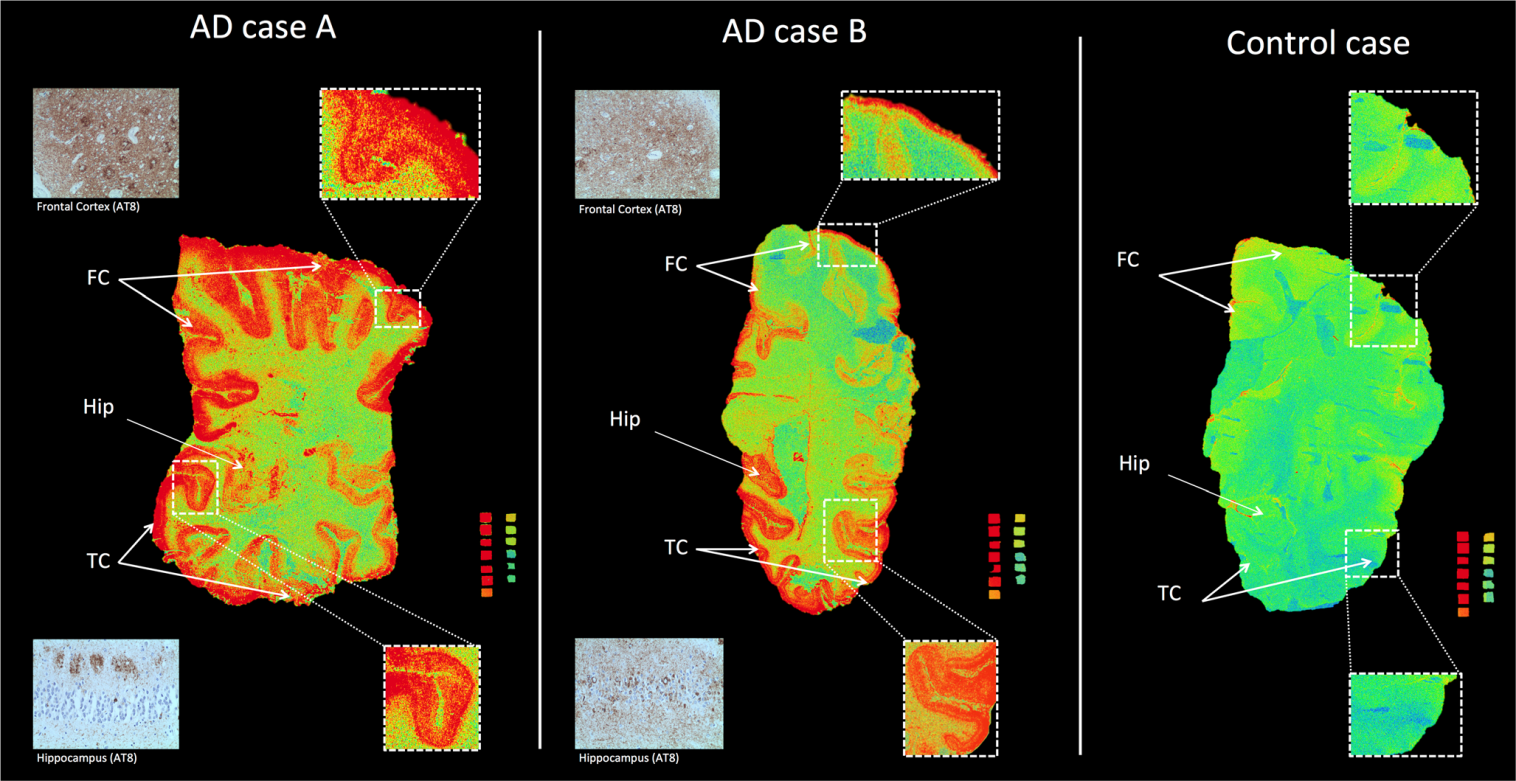
Autoradiography of ^3^H-MK6240 (1 nM) binding on frozen adjacent sections from the post-mortem left brain hemispheres of Alzheimer’s disease (AD) patients A and B and a control. The enlargements depict the cortical laminar patterns in the frontal cortex (FC) and temporal cortex (TC) lobes. AT8 immunostaining on FC and hippocampus (Hip) from a small section of the right hemisphere is also presented in the figure as a pathology reference. Red = highest binding; blue = lowest binding

The autoradiography results for the regional distribution of ^3^H-MK6240 in AD patients A (79 years old) and B (81 years old) and control (76 years old) are presented in Fig. 4. The specific binding in femtomole per milligram is shown in Table 3. We observed binding in the frontal and temporal cortices with a clear laminar pattern in tissue from AD patient A (see enlargement in Fig. 4). The distribution in tissue from AD patient B was similar but the binding was less intense, as partly explained by the different pathology (as shown in AT8 staining; Fig. 4). There was high specific binding in the entorhinal cortex from both brains (90.2 fmol/mg for patient A and 128.4 fmol/mg for patient B). Similar high specific binding was found in the fusiform gyrus (119 fmol/mg and 115.5 fmol/mg). Case A showed lower binding in the temporal cortex (49.6 fmol/mg) than case B (216.6 fmol/mg). The binding was lower for both brains in the insular cortex and was lower in the hippocampus from patient A (42.4 fmol/mg) than in that from patient B (81 fmol/mg). The inverse was seen for the frontal cortex (109.2 fmol/mg for patient A and 45.6 fmol/mg for patient B). Almost no binding was present in both total and NSP sections for the control.

**Table 3:** Specific binding of ^3^H-MK6240 (fmol/mg tissue) in regions of interest in two Alzheimer’s disease (AD) brain sections (100 μM thick)

Regions of interest	AD patient A	AD patient B
Hippocampus	42.4	81.0
Entorhinal cortex	90.2	128.4
Fusiform gyrus	119.0	115.5
Temporal cortex	49.6	216.6
Insula	24.0	32.8
Frontal cortex	109.2	45.6
Cingulate gyrus	99.9	64.9

## Discussion

The aim of this study was to characterize and compare the binding properties of ^3^H-MK6240 in human post-mortem brain tissue from AD cases with its properties in nondemented control tissue and with the properties of the first-generation tau tracers in a head-to-head comparison study.

In saturation binding assays, we observed that ^3^H-MK6240 had a high binding affinity for tissue in the temporal cortices of two AD cases: Kd was 0.32 nM and Bmax was 59.2 fmol/mg. Interestingly, when we carried out the same experiment in the parietal cortex of an EOAD brain, we observed a Kd of 0.15 nM and a Bmax of 154.7 fmol/mg. The difference in Bmax between the temporal cortex and the parietal cortex might have been the result of different brains being used in the experiment; disease progression was severe and fast for the EOAD patient.

The Bmax of ^3^H-MK6240 was similar to that published by Hostetler et al. in 2016 [21], where Bmax ranged from 7.8 nM to 93.4 nM for ^3^H-MK6240 and 15 nM to 119.7 nM for ^3^H-AV-1451. It has already been noted here that Bmax varies between brain regions. In general, the Kd of MK6240 indicates that the tracer has a higher affinity for AD tissue than the first-generation tracers THK5117 (2.2 nM) and AV-1451 (0.6–3.7) [7, 21]. THK5117 Bmax, earlier reported, seems to be higher (Bmax_1_ = 250 fmol/mg, Bmax_2_ = 1416 fmol/mg [8]) compared with MK6240 Bmax values, which probably might reflect different binding site properties and/or non-specific targets. These findings are interesting and should be taken into consideration during the use of differing brain homogenates.

In order to obtain deeper insight into the binding properties of MK6240, we carried out competitive binding assays using a wide range of unlabelled compounds. Although the saturation binding assay showed only one binding site, competitive binding studies between unlabelled MK6240 and ^3^H-MK6240 in two AD brains revealed at least two binding sites for which the tracer had different binding affinities. We saw evidence of a super-high-affinity site in the picomolar range, a high-affinity site in the nanomolar range and a low-affinity site in the higher nanomolar range. There were two binding sites for which MK6240 and AV-1451 showed affinity (one super-high and one high affinity for each tracer) and two sites for which THK5117 showed affinity (one super-high and one low affinity). The proportional analysis of these sites revealed that MK6240 had a super-high affinity for 58% of the sites. Competitive binding studies showed that both THK5117 and AV-1451 competed with ^3^H-MK6240 in a 2-site model and that a site in the picomolar range seems to be common to all the tracers. Unlabelled THK5117 competed with ^3^H-MK6240 at two sites, one with super-high affinity and one with low affinity. Unlabelled AV-1451 competed with ^3^H-MK6240 at two sites with very high affinity, one in the nanomolar range for which the affinity was higher than that of MK6240 itself. This is, to our knowledge, the first time that a compound other than the tritiated compound has competed with better affinity.

To better understand the binding properties of the different tracers at their binding sites, we analysed the fraction of binding sites targeted by these tracers while competing with the labelled compound. Although both AV-1451 and THK5117 competed with ^3^H-MK6240, AV-1451 had the strongest affinity for the ^3^H-MK6240 site. It appears that THK compounds have a high affinity for only a low percentage (37% for THK5117) of ^3^H-MK6240 binding sites (compared with 74% for AV-1451). Moreover, Ni et al. found that unlabelled AV-1451 competed with ^18^F-AV-1451 for two binding sites in the temporal cortex of AD brains, and the competitor had a high affinity for 88% of the sites. In the frontal cortex, this proportion was diminished to 61% (unpublished data). It seems that brain regions severely affected by AD contain a target displaying sites for which AV-1451 has a much higher affinity than other tracers.

**Fig. 5:**
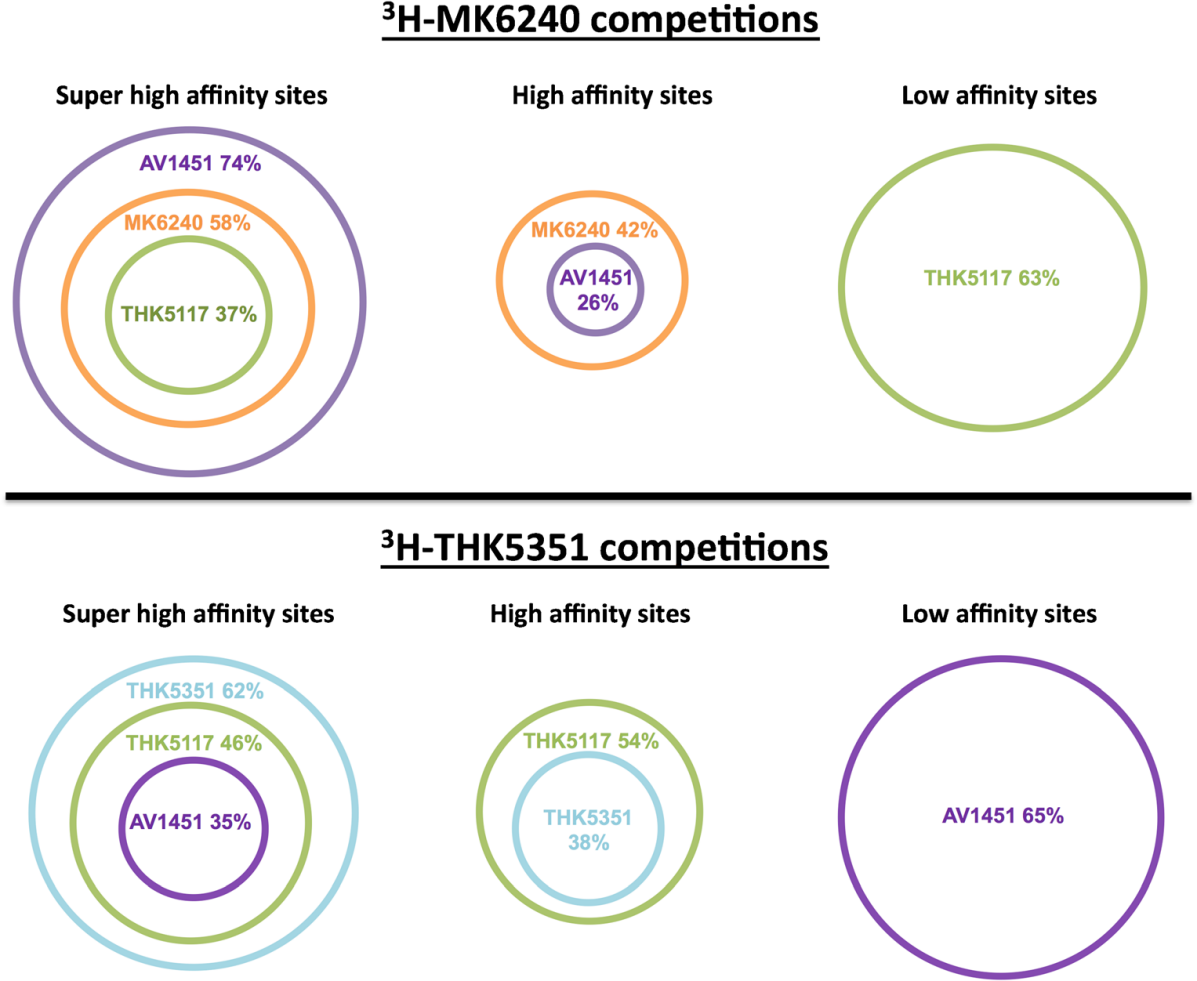
Representative schema of the percentages of the ^3^H-MK6240 and ^3^H-THK5351 binding sites for which the unlabelled competitors (MK6240, AV1451, THK5351, THK5117) had a super-high or high affinity in competitive binding studies

We have previously demonstrated that first-generation tau tracers (THK5351, THK5117 and AV-1451) share similar binding sites on tau deposits [9]. We have observed that MK6240 does not compete with ^3^H-THK5351. Hostetler et al. also reported a lack of competition between MK6240 and ^3^H-AV-1451 [21]. Interestingly, during the competition study with ^3^H-THK5351, the fraction of sites for which AV-1451 had a high affinity was 35%. AV-1451 had a high affinity for fewer ^3^H-THK5351 sites than ^3^H-MK6240 sites, which suggests that the binding properties of MK6240 are closer to those of AV-1451 than to those of THK5351. A summary of the binding properties of the tracers is presented in Fig. 5. We observed that unlabelled MK6240 and AV-1451 had a super-high affinity for a high percentage of the ^3^H-MK6240 binding sites and that unlabelled THK5117 had a low affinity for a high percentage of the ^3^H-MK6240 binding sites. Similarly, unlabelled AV-1451 had a low affinity for a high percentage of the ^3^H-THK5351 binding sites. Taken together, these competitive binding studies suggest that MK6240 is more similar to AV-1451 than to the THK compound family.

The in silico studies of AV-1451, MK6240 and THK5351 have suggested the possibility of four theoretical binding sites on tau fibrils, with a preference for one identical site BA1 for AV1451 and MK6240 and another one BA3 for THK5351 [27]; however, AV-1451 seems to have a high affinity for two binding sites. MK6240 seems to be a really specific tau PET tracer and it is possible that AV-1451 can change the conformation or the accessibility of the MK6240 specific binding site on the tau fibril, preventing ^3^H-MK6240 from binding to its target.

The regional distribution studies on brain homogenates showed that, in the temporal, frontal and parietal cortices and the hippocampus, MK6240 differentiated between AD and control tissue, with significantly increased binding to AD tissue (*p* < 0.0001) and no off-target binding in the thalamus, caudate nucleus or cerebellum (Fig. 4a). There was almost no binding to control tissue in the seven regions of interest. When we divided the samples into tissue from patients with EOAD and those with LOAD, we observed that the specific binding of ^3^H-MK6240 was significantly higher in EOAD tissue than in LOAD tissue in the cortical regions. However, our sample size was small, and it would be interesting to study the binding characteristics of ^3^H-MK6240 in a larger number of brains. Nonetheless, similar observations have been made in vivo by Schöll and colleagues [28] who showed that the retention of ^18^F-AV-1451 in cortical regions (prefrontal, premotor and inferior parietal cortices) was higher in younger patients than in older patients. A recent study using ^18^F-MK6240 also showed that 18F-MK6240 was able to discriminate between early and late stages of the disease in vivo [26]. It is not yet known whether this is the result of differences in the availability of a certain type of tau or maybe a binding site structural change.

Autoradiography ^3^H-MK6240 binding studies clearly showed a laminar binding pattern in both frontal and temporal lobes. Specific binding was elevated in the temporal cortex of AD case B. Binding in the hippocampus was lower in AD patient A than in AD patient B, which can be attributed to atrophy of the anterior part of the hippocampus, as diagnosed by magnetic resonance imaging. The difference in binding between the two samples was confirmed by AT8 pathological immunostaining. When we compared these data with previous data obtained using ^3^H-THK5117 autoradiography on tissue from the same patients, we observed that binding in the frontal cortex of patient 3 was higher than that in patient 2 (see published data [8]). This appears to suggest a difference in the binding properties of THK5117 and MK6240. There was no specific binding in tissue from the control case, who was a 76-year-old female with Braak stage I.

In conclusion, we observed that ^3^H-MK6240 had good binding properties in AD tissue, with high specific binding. First-generation tau PET tracers had at least two binding sites in common with MK6240, with binding affinities ranging from super-high to low. The binding affinity visible in PET is in the range of 10 nM; in this range, the binding of MK6240 and AV-1451 was similar but with a higher proportion of binding sites for MK6240 than for AV1451. In regional distribution studies using brain homogenates, we saw a clear difference between AD and control tissue. Almost no specific binding was seen with autoradiography in control tissue. The apparent ability of ^3^H-MK6240 to differentiate between EOAD and LOAD tissue may indicate the presence of different tau types in different stages of the disease. A larger sample size is needed to confirm this and to investigate whether it is a load of tau or the kind of tau that differs. This study highlights the complexity of the binding properties of tau tracers from the first and second developmental generations and confirms the need for deeper characterization in order to more fully understand the binding properties of tau PET tracers in AD tissue.

## Electronic supplementary material

Supplemental Fig. 1 Establishment of optimal binding assay conditions. a) Specific binding of ^3^H-MK6240 (0.5 nM; fmol/mg tissue) and b) percentage of specific binding, in increasing concentrations of post-mortem temporal cortex brain homogenates from two patients with Alzheimer´s disease (0.2, 0.5, 0.75, 1 and 2 mg/mL diluted in PBS + 0.1% BSA). Error bars represent the results from two experiments, performed in triplicate, for each of the two tissue samples. (PNG 282 kb)

High Resolution (TIFF 33972 kb)

## Data Availability

The raw data used in this study are available from the corresponding authors upon reasonable request.
