# Supplementary figures and images for: Characterization of MK6240, a tau PET tracer, in autopsy brain tissue from Alzheimer’s disease cases
Source: Eur J Nucl Med Mol Imaging. 2020 Sep 24;48(4):1093–102. doi: 10.1007/s00259-020-05035-y (PMC8041708; doi:10.1007/s00259-020-05035-y)

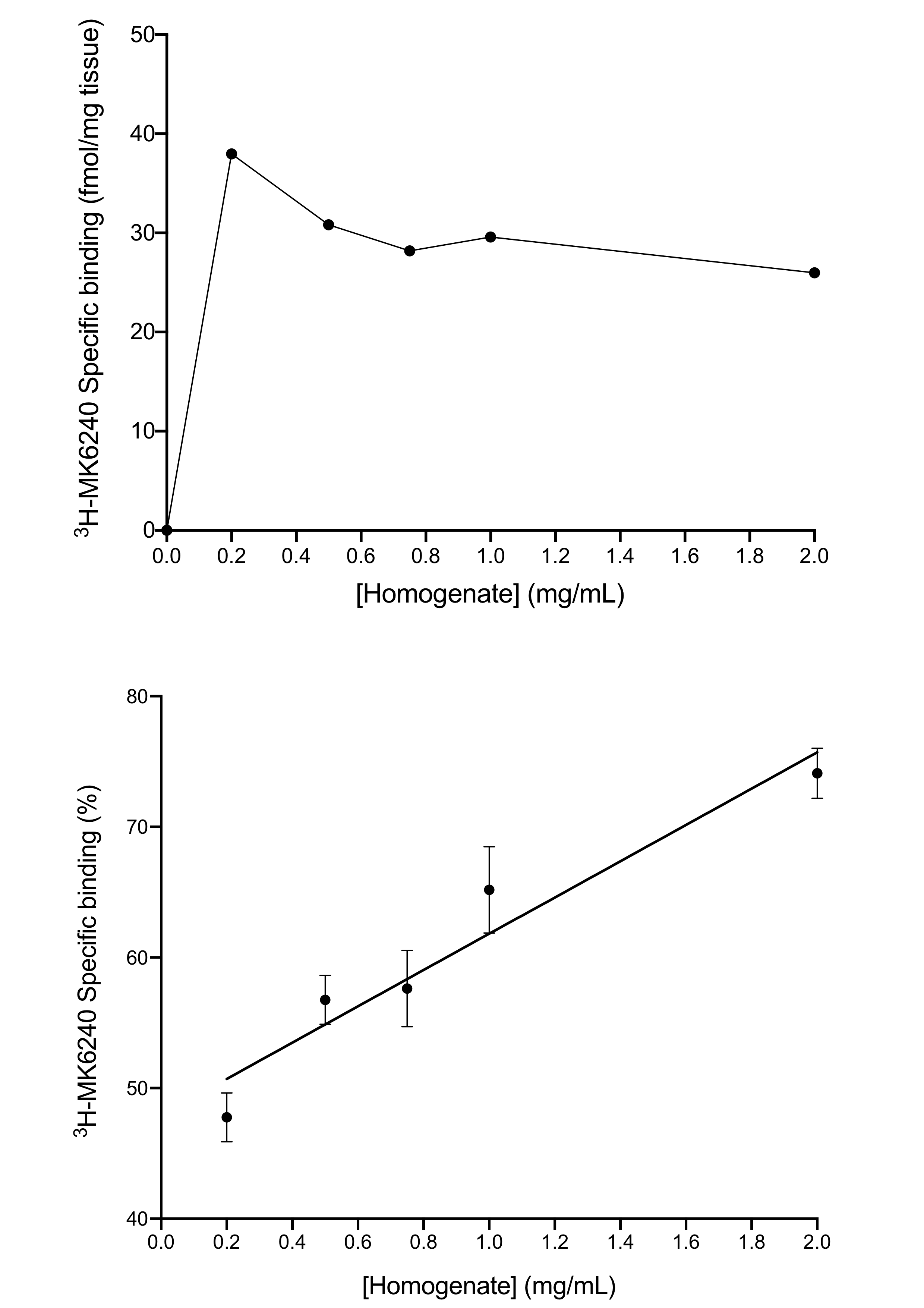

Supplement: Supplementary file 1 — Establishment of optimal binding assay conditions. a) Specific binding of 3H-MK6240 (0.5 nM; fmol/mg tissue) and b) percentage of specific binding, in increasing concentrations of post-mortem temporal cortex brain homogenates from two patients with Alzheimer´s disease (0.2, 0.5, 0.75, 1 and 2 mg/mL diluted in PBS + 0.1% BSA). Error bars represent the results from two experiments, performed in triplicate, for each of the two tissue samples. (PNG 282 kb) [file 259_2020_5035_Fig6_ESM.png]

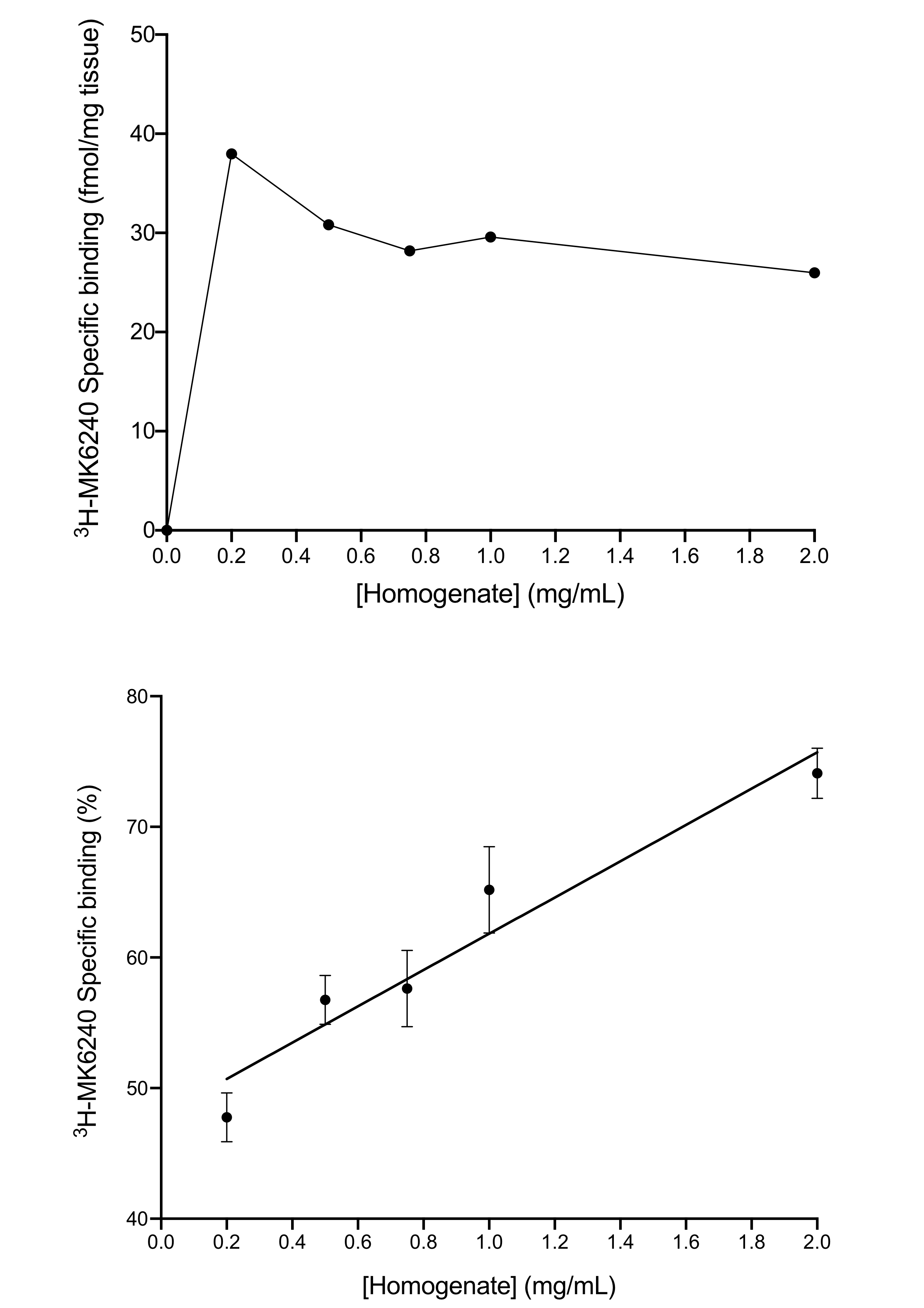

Supplement: Supplementary file 2 — High Resolution (TIFF 33972 kb) [file 259_2020_5035_MOESM1_ESM.tiff]
